# Higher Numbers of Pregnancies Associated With an Increased Prevalence of Gestational Diabetes Mellitus: Results From the Healthy Baby Cohort Study

**DOI:** 10.2188/jea.JE20180245

**Published:** 2020-05-05

**Authors:** Bingqing Liu, Lulu Song, Lina Zhang, Lulin Wang, Mingyang Wu, Shunqing Xu, Zhongqiang Cao, Youjie Wang

**Affiliations:** 1 Department of Maternal and Child Health, School of Public Health, Tongji Medical College, Huazhong University of Science and Technology, Hubei, China; 2 Key Laboratory of Environment and Health, Ministry of Education & Ministry of Environmental Protection, and State Key Laboratory of Environmental Health, School of Public Health, Tongji Medical College, Huazhong University of Science and Technology, Hubei, China; 3 Wuhan Children’s Hospital (Wuhan Maternal and Child Healthcare Hospital), Tongji Medical College, Huazhong University of Science and Technology, Hubei, China

**Keywords:** gestational diabetes mellitus, number of pregnancy, cohort study

## Abstract

**Background:**

Pregnancy leads to substantial maternal metabolic and lifestyle alterations. However, it is still unclear whether repeated exposure to these changes will influence the development of gestational diabetes mellitus (GDM). In the present study, we aimed to investigate the association between the number of pregnancies and GDM among Chinese women.

**Methods:**

A total of 7,008 subjects from the Healthy Baby Cohort study were included in this study. The number of pregnancies was classified into three categories: 1, 2, or ≥3 pregnancies. GDM was diagnosed using International Association of Diabetes and Pregnancy Study Groups criteria. Multivariate logistic regression models were used.

**Results:**

In the fully adjusted model, women with ≥3 pregnancies had a 1.27-fold (95% confidence interval [CI], 1.05–1.54) higher risk of GDM. Among women ≥30 years old, 2 and ≥3 pregnancies were associated with a higher risk of GDM (odds ratio [OR] 1.32; 95% CI, 1.01–1.73 and OR 1.54; 95% CI, 1.17–2.01, respectively). Among women with a pre-pregnancy BMI <24 kg/m^2^, ≥3 pregnancies were associated with a 1.35-fold (95% CI, 1.09–1.67) higher risk of GDM.

**Conclusions:**

Our findings suggested that higher numbers of pregnancies is an independent risk factor of GDM. The association between number of pregnancies and GDM was more prominent among women who were ≥30 years old or with a pre-pregnancy BMI <24 kg/m^2^.

## INTRODUCTION

Gestational diabetes mellitus (GDM) is defined as any degree of glucose intolerance that begins or is recognized for the first-time during pregnancy.^1^ According to the International Association of Diabetes and Pregnancy Study Groups (IADPSG) criteria, GDM constitutes 17.8% (range, 9.3–25.5%) of all pregnancies^2^ and has long-lasting health consequences for both mothers and their offspring. GDM increases the maternal risk of developing diabetes, and the offspring’s risk of macrosomia, obesity, glucose intolerance, and diabetes.^3^^,^^4^ Thus, it is important to identify women at risk of GDM, who would benefit from early preventative strategies. Previously, several GDM risk factors have been identified, including increasing maternal age, obesity, and a family history of diabetes.^1^ However, notable gaps remain in our understanding of risk factors and the pathogenesis of GDM.

Pregnancy leads to substantial maternal metabolic and lifestyle alterations, including decreased insulin sensitivity, an accumulation of body fat, body fat central redistribution, decreased physical activity, and increased calorie intake,^5^^–^^7^ which are associated with higher risk of GDM.^8^^,^^9^ However, it is still unclear whether repeated exposure to these metabolic and lifestyle changes influences the development of GDM in future pregnancies. Various studies have investigated the association between parity and GDM, with conflicting results.^10^^–^^13^ However, none of these studies took abortion or stillbirth into consideration. Spontaneous miscarriage occurs in approximately 15% of clinically recognized pregnancies.^14^ In China, approximately 10 million women received an induced abortion in 2015,^15^ and approximately 50% of women who underwent an induced abortion experienced repeated abortions.^16^ Therefore, a significant proportion of women might have a high number of pregnancies, despite having a relatively low parity. Because the metabolic alterations caused by pregnancy were observed as early as the first trimester,^17^ abortions and stillbirth might also be accompanied by metabolic alterations lead by pregnancy. Thus, it is necessary to investigate whether a high number of pregnancies is associated with GDM risk.

In the present study, we investigated the association between the number of pregnancies and GDM among Chinese women. Our hypothesis was that women with a higher number of pregnancies were at higher risk of developing GDM than women with one pregnancy.

## MATERIAL AND METHODS

### Participants

The study subjects were from Healthy-Baby Cohort-Wuhan (HBC-Wuhan), which is a prospective cohort conducted to explore the environmental and genetic factors that affect health and development. This study recruited 11,311 pregnant women, who came for their first antenatal care visit or birth delivery at Wuhan Children’s Hospital (Wuhan Maternal and Child Healthcare Hospital), from September 2012 to October 2014. After excluding those women with a history of diabetes (*n* = 6) and had no available information on fasting, 1-h, or 2-h plasma glucose data (*n* = 4,297), 7,008 women were included in the final analysis.

This study was approved by the Medical Ethics Committee of School of Public Health, Tongji Medical School and the Wuhan Children’s Hospital (Wuhan Maternal and Child Healthcare Hospital). Signed informed consent was obtained from all participants.

### Assessment of GDM

Participants underwent a 75-g oral glucose tolerance test (OGTT) at 24–28 weeks of gestation. Plasma glucose levels were measured using the Roche Modular P800 automated biochemistry analyzer (Roche Diagnostics USA, Indianapolis, IN, USA) at the Wuhan Children’s Hospital (Wuhan Maternal and Child Healthcare Hospital). GDM was diagnosed using the diagnostic criterion of the IADPSG.^18^ Women whose 75g-OGTT plasma glucose level met one or more of the following criterions were defined as having GDM: fasting plasma glucose level ≥5.1 mmol/L; 1-h plasma glucose level ≥10.0 mmol/L; 2-h plasma glucose level ≥8.5 mmol/L.

### Assessment of pregnancy

Information on the number of pregnancies was extracted from medical record of Wuhan Children’s Hospital (Wuhan Maternal and Child Healthcare Hospital). Number of pregnancies was classified into three categories: 1, 2, or ≥3 pregnancies.

### Assessment of covariates

In this study, we collected demographic information (age, annual household income, and education background) and lifestyle information (cigarette smoking, passive smoking exposure, and alcohol drinking) via structured questionnaire. All the participants were interviewed face-to-face by trained nurses during delivery hospitalization. Pre-pregnancy weight was self-reported at the first antenatal care visit (at the first trimester), while measured standing height and the results of laboratory were abstracted from medical records. Pre-pregnancy body mass index (BMI) was calculated as weight (kg) divided by the height squared (m^2^).

### Statistical analysis

Categorical and numerical variables were summarized as proportion (%) and means (standard deviation [SD]), respectively. Differences in means and proportions between GDM and non-GDM groups were analyzed with analysis of variance (ANOVA) for numerical variables and Chi-square test for categorical variables. A series of logistic regression models were employed to assess the association between number of pregnancies and GDM. Women with one pregnancy were used as the reference group. Model 1 was used to summarize the crude odds ratios without adjustment of any covariates. Model 2 was adjusted for pre-pregnancy BMI. Model 3 was the fully-adjusted model, in which we adjusted maternal age (continuous), pre-pregnancy BMI (continuous), education (middle school or below, high school, or college or above), annual household income (<50,000, 50,000–100,000, or ≥100,000 Yuan), smoking status (current/ever smoker or never smoker), drinking status (yes or no), and passive smoking (yes or no).

Because increasing age and pre-pregnancy BMI were acknowledged risk factors of GDM,^1^ we further employed subgroup analysis to assess the potential effect modifications by age and pre-pregnancy BMI. SAS 9.4 (SAS Institute Inc, Cary, NC, USA) was used for all analysis. Statistical significance were determined using a two-side probability set at *P* < 0.05. For the test of interactive effect, statistical significance was set at a two-side probability of *P* < 0.1.^19^

## RESULTS

Characteristics of the participants are shown in Table 1. Among 7,008 participants, a total of 1,030 women (14.7%) were diagnosed as having GDM based on the diagnostic criterion suggested by IADPSG. Women had GDM were significantly older (*P* < 0.001), fatter before pregnancy (*P* < 0.001), and and had lower education background (*P* < 0.001).

**Table 1.** Characteristics of the participants according to gestational diabetes

Characteristics	GDM		*P*

	Yes	No	
	*N* = 1,030	*N* = 5,978	
Age, years, mean (SD)	29.6 (3.9)	28.3 (3.4)	<0.001
Pre-pregnancy BMI, kg/m^2^, mean (SD)	21.5 (3.1)	20.6 (2.7)	<0.001
Education			
Middle school or below (%)	132 (12.8)	514 (8.6)	<0.001
High school (%)	193 (18.7)	1012 (16.9)	
College or above (%)	705 (68.4)	4451 (74.5)	
Annual household income (Yuan)^*^			
<50,000 (%)	421 (41.9)	2311 (39.3)	0.130
50,000–100,000 (%)	417 (41.5)	2459 (41.8)	
>100,000 (%)	167 (16.6)	1117 (19.0)	
Cigarette smoking status			
Current or ever smokers (%)	9 (0.9)	30 (0.5)	0.138
Never smokers (%)	1021 (99.1)	5948 (99.5)	
Passive smoking exposure			
Yes (%)	211 (20.5)	1274 (21.3)	0.549
No (%)	819 (79.5)	4704 (78.7)	
Alcohol drinking			
Yes (%)	22 (2.1)	129 (2.2)	0.964
No (%)	1008 (97.9)	5849 (97.8)	

BMI, body mass index; GDM, gestational diabetes mellitus; SD, standard deviation.

^*^One Yuan RMB is equal to 0.1462 United States dollars.

We used three logistic regression models to assess the associations between number of pregnancy and GDM. In the unadjusted model, women who had 2 and ≥3 pregnancies had 1.29 (95% CI, 1.10–1.51) and 1.89 (95% CI, 1.60–2.23) times higher risk of GDM than women who had one pregnancy. After adjusting for age, pre-pregnancy BMI, education, annual household income, smoking status, drinking status, and passive smoking exposure, we found that ≥3 pregnancies was still associated with higher risk of GDM (OR 1.27; 95% CI, 1.05–1.54).

**Figure 1.**
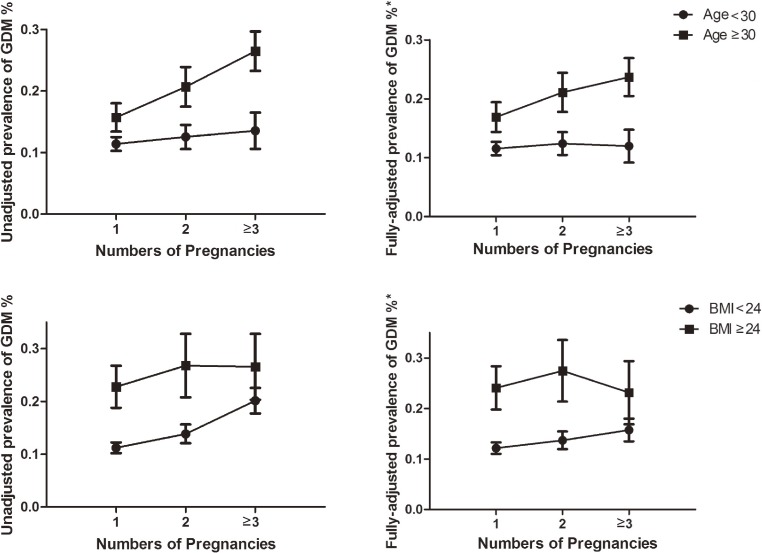
Prevalence of gestational diabetes in different age and pre-pregnancy body mass index groups according to number of pregnancies. ^*^The adjusted covariates included age, pre-pregnancy BMI, education, annual household income, smoking status, drinking status and passive smoking exposure.

We further did stratified analysis by age and pre-pregnancy BMI. In the fully-adjusted model, 2 and ≥3 pregnancies were associated with higher risk of GDM (OR 1.32; 95% CI, 1.01–1.73 and OR 1.54; 95% CI, 1.17–2.01, respectively) in women ≥30 years old (*P*_for interaction_ = 0.048; Figure 1 and Table 2). After adjusted potential confounders, ≥3 pregnancies were associated with higher risk of gestational diabetes (OR 1.35; 95% CI, 1.09–1.67) in women with pre-pregnancy BMI <24 kg/m^2^ (Figure 1 and Table 2). However, we did not find significant interactions between pre-pregnancy BMI and number of pregnancy on GDM (*P*_for interaction_ = 0.591).

**Table 2.** Odd Ratios and 95% confidence intervals for GDM according to number of pregnancies

Number of pregnancy	Number of subjects	Model 1: unadjusted	Model 2: model 1+BMI	Model 3: fully-adjusted model^*^
Total population				
1	4,060	1	1	1
2	1,698	1.29 (1.10, 1.51)	1.27 (1.08, 1.49)	1.16 (0.98, 1.37)
≥3	1,250	1.89 (1.60, 2.23)	1.79 (1.51, 2.11)	1.27 (1.05, 1.54)
Age <30 years				
1	3,091	1	1	1
2	1,093	1.12 (0.90, 1.38)	1.11 (0.90, 1.38)	1.08 (0.87, 1.35)
≥3	517	1.22 (0.93, 1.61)	1.19 (0.90, 1.57)	1.04 (0.77, 1.39)
Age ≥30 years				
1	969	1	1	1
2	605	1.40 (1.08, 1.82)	1.38 (1.06, 1.80)	1.32 (1.01, 1.73)
≥3	733	1.94 (1.52, 2.46)	1.89 (1.49, 2.40)	1.54 (1.17, 2.01)
BMI <24 kg/m^2^				
1	3631	1	1	1
2	1487	1.27 (1.06, 1.53)	1.27 (1.06, 1.52)	1.15 (0.95, 1.38)
≥3	1058	2.00 (1.66, 2.40)	1.94 (1.62, 2.33)	1.35 (1.09, 1.67)
BMI ≥24 kg/m^2^				
1	426	1	1	1
2	209	1.24 (0.85, 1.82)	1.25 (0.85, 1.83)	1.20 (0.81, 1.78)
≥3	192	1.23 (0.83, 1.82)	1.23 (0.83, 1.82)	0.95 (0.60, 1.50)

BMI, body mass index.

^*^fully-adjusted model: adjusted for age, pre-pregnancy BMI, education background, annual household income, smoking status, drinking status and passive smoking exposure.

We compared the main characteristics between women who were included and excluded. Except for drinking status, the main characteristics of the included and excluded participants, including number of pregnancy (1.7 vs. 1.9), age at delivery (28.5 vs. 27.8 years), pre-pregnancy BMI (20.7 vs. 20.3 kg/m^2^), gestational weight gain (17.3 vs. 16.9 kg), and passive smoking exposure (21.2% vs. 23.9%) were statistically different.

## DISCUSSION

In this study, we found that women with ≥3 pregnancies had a 1.27-fold (95% CI, 1.05–1.54) higher risk of developing GDM, while 2 pregnancies was not associated with GDM. These associations were more prominent among women aged ≥30 years old or women with pre-pregnancy BMI <24 kg/m^2^.

There have been few studies on the association between the number of pregnancies and GDM, some of them have conflicting results. A study conducted among 11,205 women found that ≥3 live births increased the risk of GDM in white, black, and Southeast Asian women.^12^ Another study found that multiparity was associated with a higher risk of GDM.^13^ These two studies support our current findings. Inconsistent with these two studies, Xiong et al found that multiparity was not associated with GDM.^11^ It should be noted that these studies used different criteria to diagnose GDM, which might at least partially explain the discrepancy in the results. Moreover, these studies only investigated the association between the number of live births and GDM, while abortions and stillbirth, which are also accompanied by metabolic alterations lead by pregnancy,^17^ were not considered.

The mechanism underlying the link between the number of pregnancies and GDM is unclear. During pregnancy, the increased secretion of steroids and peptide hormones leads to a progressive rise in maternal tissue insulin resistance.^6^ Although glucose homeostasis is restored to preconception levels shortly after delivery,^20^ repeated exposure to these drastic hormonal and metabolic changes may still pathologically perturb glucose metabolism. Lifestyle changes caused by pregnancy may also increase the risk of GDM among women with a high number of pregnancies. Pregnant women tend to reduce their physical activity and increase their calorie intake during and after pregnancy,^5^^,^^21^^,^^22^ especially in China, where women are encouraged to rest in bed and consume high-calorie food during pregnancy and the postpartum period.^23^ It is well established that a high-calorie diet induces insulin resistance, while physical inactivity before and during pregnancy is associated with GDM.^24^^,^^25^ These unhealthy changes in lifestyle may have long-term metabolic effects that extend into future pregnancies. Besides, those pregnancy-related unhealthy lifestyles might increase gestational weight gain during the next pregnancy, which is a risk factor of GDM,^26^ leading to the hypothesis that the association between number of pregnancy and GDM might be mediated by gestational weight gain. However, gestational weight gain was not significantly different among pregnant women with 1, 2, or ≥3 pregnancies in the present study (*P* = 0.427), which suggests that gestational weight gain was not a mediator for the relationship between number of pregnancy and GDM. Thus, gestational weight gain could probably not account for the observed positive association between the number of pregnancies and GDM in the current study.

In this study, we found that the association between the number of pregnancies and GDM was more prominent among women aged ≥30 years. The rate of impaired glucose tolerance and impaired fasting glucose was previously found to increase with age among women.^27^ This could be attributed to the reduced physical activity and muscle mass reported in older women.^28^^,^^29^ Physical inactivity leads to a decline in glucose tolerance and an increase in fasting blood glucose,^30^^,^^31^ while skeletal muscle mass was reported to negatively predict 2-hour plasma glucose levels in subjects with normal glucose tolerance.^32^ These age-related changes in glucose metabolism would be exacerbated by pregnancy, which may explain the stronger association between the number of pregnancies and GDM among women aged ≥30 years.

We also found that, although overweight/obesity women had higher GDM prevalence, the association between the number of pregnancies and GDM was more prominent among lean women. However, it should be noted that the interaction between pre-pregnancy BMI and number of pregnancies on GDM was not significant, which indicated that this BMI-specific association between number of pregnancies and GDM might be attributed to chance. The pre-pregnancy BMI-stratified analyses should be considered as exploratory. It’s reported that obese women demonstrate less weight gain and smaller rises in fasting plasma glucose during pregnancy.^33^ Thus, pregnancies appear to have less effect on glucose homeostasis in obese women. Therefore, although the obesity/overweight women had higher GDM prevalence, the association between the number of pregnancies and GDM was not significant.

The present study has several strengths. It had a relatively large sample size, and OGTT measurements were performed using the same instruments at one hospital, which decreased detection bias. However, it has a number of limitations. First, the relatively large number of subjects excluded from the study might have introduced selection bias. However, although there were significant differences between subjects who were included and excluded, the differences in major variables between the two groups were relatively small. Because of the large sample of the inclusion and exclusion, a slight difference would lead to a statistically significant difference. Second, number of pregnancies was obtained from medical records, but it was self-reported to doctors during the first prenatal visit. Therefore, recall bias might have occurred. Moreover, information about abortions and stillbirths is considered private in China, so the numbers of these pregnancies might be under-reported, which might lead to an under-estimation of the association between the number of pregnancies and GDM. Third, our study participants were all Chinese women, which minimized the confounding effects by ethnic background but might reduce the extension of our results to non-Asian populations. Fourth, we cannot control for unmeasured confounding, such as family history of diabetes and diet. Further studies with this information are needed to confirm the finding of this study.

In summary, we found that higher number of pregnancies was independently associated with a higher risk of GDM, particularly among women aged ≥30 years old and women with pre-pregnancy BMI <24 kg/m^2^. These findings suggest that clinical practitioners should take the number of pregnancies into consideration while assessing GDM risk. Further prospective studies are needed to confirm our findings and to elucidate the mechanism underlying the association between the number of pregnancies and GDM.
